# Discrimination Experiences among Asian American and Pacific Islander Adults during the COVID-19 Pandemic and Their Association with Mental Health Outcomes: Updated Findings from the COMPASS Study

**DOI:** 10.3390/ijerph21060799

**Published:** 2024-06-19

**Authors:** Marcelle M. Dougan, Marian Tzuang, Bora Nam, Oanh L. Meyer, Janice Y. Tsoh, Van M. Ta Park

**Affiliations:** 1 Department of Public Health and Recreation, San José State University, San Jose, CA 95192, USA; 2 Department of Community Health Systems, School of Nursing, University of California-San Francisco, San Francisco, CA 94143, USA; yuan.tzuang@ucsf.edu (M.T.); bora.nam@ucsf.edu (B.N.); van.park@ucsf.edu (V.M.T.P.); 3 Department of Neurology, School of Medicine, University of California, Davis (UCD), Sacramento, CA 95817, USA; olmeyer@ucdavis.edu; 4 Asian American Research Center on Health (ARCH), University of California-San Francisco, San Francisco, CA 94143, USA; janice.tsoh@ucsf.edu; 5 Department of Psychiatry and Behavioral Sciences, School of Medicine, University of California-San Francisco, San Francisco, CA 94143, USA

**Keywords:** pandemic, discrimination, mental health, AAPI, AANHPI, COVID-19

## Abstract

Background: Reports of escalated discrimination experiences among Asian American and Native Hawaiian Pacific Islanders (AANHPI) continue. Methods: Using the original and follow-up surveys of the COVID-19 Effects on the Mental and Physical Health of AAPI (Asian American and Pacific Islanders) Survey Study (COMPASS I and COMPASS II) (n = 3177), we examined changes over approximately a 1-year period in discrimination experiences attributable to being AAPI and factors associated with worse mental health outcomes. Results: Experiences of discrimination remained high in COMPASS II with 60.6% (of participants (compared to 60.2% among the same people in COMPASS I) reporting one or more discrimination experiences, and 28.6% reporting worse mental health outcomes. Experiences of discrimination were associated with modest but significant increase in the odds of worse mental health: adjusted OR 1.02 (95% CI 1.01–1.04). Being younger, being of Native Hawaiian/Pacific Islander or Hmong descent (relative to Asian Indian), and having spent 50% or less of their lifetime in the US (vs. US born), were significantly associated with worse mental health. Conclusions: The fall-out from the pandemic continues to adversely impact AANHPI communities. These findings may help influence policy initiatives to mitigate its effects and support interventions designed to improve mental health outcomes.

## 1. Introduction

Reports of escalated discrimination/xenophobia among Asian American and Native Hawaiian/Pacific Islander (AANHPI) populations in the United States (US) during the COVID-19 pandemic are alarming. California is home to the largest AANHPI population in the US [1]. Even before the COVID-19 pandemic, there were already reports of higher rates of racial discrimination among AANHPI Californians (17%) compared to Californians overall (12%) in the workplace [2]. Moreover, there were significant differences in rates of racial discriminatory experience by AANHPI subgroups with Native Hawaiian and Pacific Islander (NHPI) (29%), Asian Indian (24%), and Hmong (19%) Californians reported higher rates of racial discrimination within this subgroup [2].

Since the COVID-19 pandemic, AANHPI individuals have been victims of social stigma, racist incidents, and hate crimes related to COVID-19 [3,4]. A poll of 1001 US adults in April 2020 reported 32% of the respondents have witnessed Asian people being blamed for the pandemic, and among Asian respondents, 60% witnessed the same problem [5]. The Stop AAPI Hate website received nearly 1500 reports of COVID-19 related discrimination against AAPI within the first four weeks of the pandemic despite shelter-in-place (SIP) orders having been implemented across many parts of the country [6]. A Stop AAPI Hate report found 11,409 hate acts occurred between January 2020 and December 2022, with younger individuals (under 18 years old) reporting higher percentages of written, visual, or auditory forms of harassment and those 60 years and older reporting higher percentages of being physically harmed. Over half (51%) of the hate acts occurred in public spaces and businesses (28%), such as restaurants or supermarkets; however, as the age of respondents increased, there was a higher likelihood of hate incidents happening in a private residence with increasing age of the respondents [7]. Correspondingly, the economic consequences of anti-Asian racism as a result of the COVID-19 were also significant, translating to more than 7 billion US dollars in lost revenue for Asian restaurants in 2020 alone [8].

Existing data or incident reports of COVID-19 related discrimination were mostly limited to English-speaking respondents. Preliminary findings from the **C**OVID-19 Effects **O**n the **M**ental and **P**hysical Health of **A**sian American and Pacific Islanders **S**urvey **S**tudy (COMPASS I), a survey of a convenient national sample of >4900 AANHPI adults conducted between October 2020 to February 2021 offered insights, for the first time, into the discrimination experiences from AANHPI participants nationwide (with nearly 30% of the participants completing the survey in a language other than English). Three out of five participants reported experiencing discrimination in the past 6 months, and 41% said they had experienced some (mild/moderate/severe) changes in their experience with racial discrimination [9]. The proportions of COMPASS I participants who “strongly or somewhat agreed” to the following statements about how the COVID-19 pandemic is affecting people of their race/ethnicity in the US were as follows: 59.0% believed that the country had become more dangerous; 40.6% believed that most social/mass media reports about COVID-19 created bias against their race/ethnicity; 39.05% saw a lot more cyberbullying; 34.1% worried about people thinking they had COVID-19; 31.5% believed that they were more likely to lose their job; 15.3% believed that they would not receive COVID-19 healthcare as good as others; and, 10.6% believed they were more likely to get COVID-19 [9]. 

A strong body of evidence suggests that experiences of discrimination can lead to adverse mental health outcomes [10,11]. However, less is known about the impact of discrimination on the mental health of AANHPI populations, particularly in light of the pandemic. These populations are particularly vulnerable as some, for example, South Asians, are at higher odds of experiencing psychological distress [12]. Notably, the overall utilization of services among this population is also low, and may vary by factors including gender and English language proficiency [13]. As such, mental health disorders can be under-diagnosed in these groups [14]. Although there have been some studies addressing this question, many of these are cross-sectional, limiting our understanding of the medium- and longer-term mental health effects of discrimination [15]. Further, there is also considerable variation in the mental health status of AANHPI groups. For example, a 2018 report [16]. stated that 78% of Filipino American women rate their mental health as very good or excellent compared to 45% of Chinese American women and 50% of Vietnamese American women, while among men, Japanese Americans and Korean Americans experience higher rates of suicide compared to other men in this sub-group. 

COMPASS II, a follow-up survey conducted approximately 12 months afterwards, provided an unprecedented opportunity to further understand the longitudinal impacts of COVID-19 related discrimination on mental health experienced by AANHPI individuals that were inclusive of the experiences from non-English speakers. 

Therefore, we prospectively examined the relationship between everyday experiences of discrimination and change in mental health over approximately a one-year period. We hypothesized that experiences of discrimination would be associated with worse mental health outcomes over time. We also examined whether there were any differences in the effect of discrimination on mental health by cultural group, age, sex, education, and nativity. We hypothesized that there would be differences by these factors on the relationship between discrimination and mental health.

## 2. Materials and Methods

### 2.1. Study Eligibility, Recruitment and Procedures

All subjects gave their informed consent for inclusion before they participated in the study. The study was conducted in accordance with the Declaration of Helsinki, and the protocol was approved by University of California San Francisco Institutional Review Board (Protocol #: 20-31925). 

The original study, COMPASS (now referred to as COMPASS I) is a national, cross-sectional, community-based survey that was instituted to examine the effects of the COVID-19 pandemic on AANHPIs. The eligibility and recruitment criteria have previously been described [9]. In the original cohort, a total of 5418 respondents agreed to participate in the study, and data were collected from October 2020 through May 2021. Approximately one year after COMPASS I was initiated, a follow-up study (COMPASS II) was conducted from December 2021 through May 2022, to examine the changes over time. A total of 5359 participants who remained eligible and provided contact information were invited to participate in the follow-up study. Initially, email invitations were sent, followed by five reminder emails. Additionally, two follow-up reminders were sent to participants who partially completed the survey. For those who did not have personal email addresses (640 participants) and those who completed COMPASS I with the assistance of others, including research staff and family members (551 participants), a separate individual outreach effort was conducted via phone with the help of community partners. Among those contacted, 64.3% agreed to participate in the follow-up study (COMPASS II; n = 3444). In COMPASS II, 88.3% participants self- completed the survey. For the present study, we excluded those who were missing at least one response to any of the discrimination questions (n = 181; 5.2%), or at least one of the responses to the PHQ-4 questions (n = 86; 2.6%). The final dataset comprised 3177 participants. The Research Electronic Data Capture [17,18] hosted at the University of California, San Francisco was used to collect the data. The World Health Organization’s process of translation and adaptation of instruments [19] was used to guide the translations of the study materials that were not already available in the targeted language(s).

### 2.2. Measurement Framework

**Figure 1:**
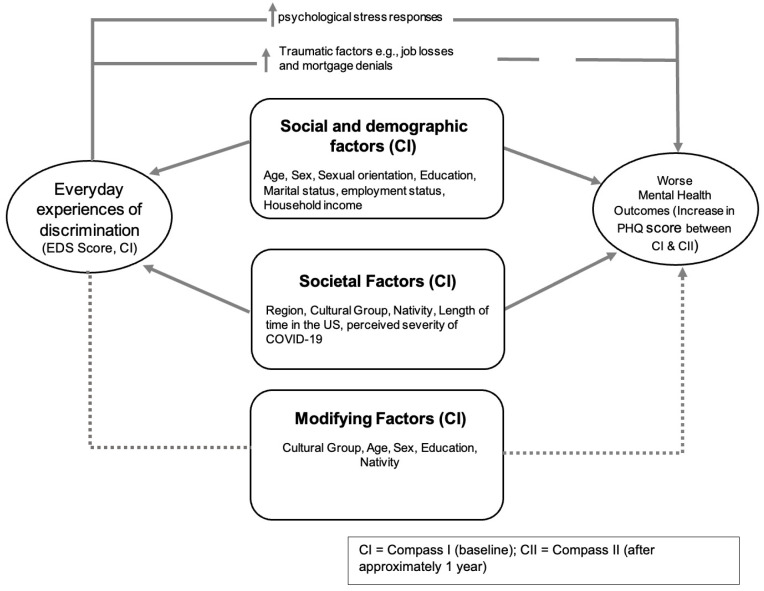
Conceptual framework for relationship between everyday experiences of discrimination and mental health.

This study’s measurement framework, guided by the previously discussed literature and the National Institute of Minority Health and Health Disparities (NIMHD) Research Framework [20], examined multi-level influences on discrimination experiences, which operate within the sociodemographic contexts of AANHPIs (e.g., cultural group, age, sex, sexual orientation, education, income, English proficiency, nativity, years in the US) that cut across individual, interpersonal (marital status, employment status), and community (geographic regions, perceived severity of COVID-19) levels that are modifiable and COVID-specific. A conceptual framework that describes the inter-relatedness of these variables in relation to mental health is shown in Figure 1 below. As depicted, everyday experiences of discrimination can impact mental health outcomes either through increased physiological stress responses or through the presence of more traumatic factors such as job losses or mortgage denials. In addition, societal factors and sociodemographic factors may affect or modify this association.

### 2.3. Measures

Mental Health. Mental health was assessed using the PHQ-4 [21] in both surveys assessing four depression and anxiety symptoms experienced over the past two weeks: (1) feeling nervous, anxious, or on edge, (2) not being able to stop or control worrying, (3) little interest or pleasure in doing things, and (4) feeling down, depressed, or hopeless. Responses were provided on a 4-point scale: 0: not at all, 1: several days, 2: more than half the days, 3: nearly every day.

Discrimination experiences during COVID-19 pandemic. Perceived discriminatory experience was assessed by using a revised 8-item Everyday Discrimination Scale (EDS) [22,23]. Participants were asked, “In the past 6 months, how often have…” with each of the 8 items ending with “because you are Asian, Asian American or Pacific Islander”. The 8 items were:

1. …you been treated with less respect than other people…
2. …you been treated unfairly at restaurants or stores…
3. …people criticized your accent or the way you speak…
4. …people acted as if they think you are not smart…
5. …people acted as if they are afraid of you…
6. …people acted as if they think you are dishonest…
7. …people acted as if they’re better than you are…
8. …you been threatened or harassed…

We used the “past 6 months” for COMPASS (original EDS used “12 months”) to measure the occurrence of discrimination experienced within the duration of the COVID-19 pandemic using a 4-point scale: 0 (never), 1 (rarely), 2 (sometimes), and 3 (often). The EDS is widely used with high reliability [22,24,25] and construct validity, [26,27] and has been studied in different racial/ethnic groups, including Asian Americans, with adequate psychometric properties [22,28,29,30,31,32]. However, we acknowledge that these studies have primarily focused on certain AANHPI populations (e.g., Chinese, Vietnamese), and the EDS has not been sufficiently validated with other AANHPI groups (e.g., South Asians, NHPI). The standardized Cronbach alpha for the EDS in this study was 0.92, suggesting very good reliability for this population. All covariates were selected because of their hypothesized relationships with both discrimination experiences and mental health.

Sociodemographic characteristics: Participants’ demographic information were drawn from CARE’s Socio-Demographics Survey, [33] and thus translated into all target languages. Participants were asked about their race, ethnic/cultural group, sex, sexual orientation, year of birth, nativity, years lived in the US, employment, education, and annual household income in 2019. 

Interpersonal level characteristics. Marital status and employment status were again drawn from CARE’s Socio-demographics Survey [33]. 

Community level characteristics. 

US geographic region was obtained per the US Census Bureau’s definition of region (Midwest/Northeast/South/West) [34]. by converting the zip code, or internet protocol address in the case of missing zip codes (n = 146).

Perceived severity of COVID-19 was developed by COMPASS. Participants were asked, “How would you rate the severity of COVID-19 pandemic at where you live in comparison to other locations in the US?” Responses were recorded on a 5-point Likert scale (1 = a lot less severe than most other places in the US, 2 = somewhat less severe, 3 = about the same, 4 = somewhat more severe), 5 = a lot more severe).

### 2.4. Statistical Analysis

Descriptive statistics were assessed using means and standard deviations in addition to the range for continuous variables, and numbers and percentages for categorical variables. The outcome variable for this study was worse depression/anxiety symptoms, “worse mental health” over time. Using the responses on the PHQ-4 scale, participants’ individual scores across all 4 questions were summed up for each of the two surveys. Then, a difference was obtained by subtracting the COMPASS I score from COMPASS II score. “Worse mental health” was then modeled as a binary variable, with participants having a positive difference (i.e., their scores increased between COMPASS I and COMPASS II) were classified as having worse mental health (“yes”), and those who had a null or negative difference (i.e., their scores did not change or decreased between COMPASS I and COMPASS II) were classified as not (“no”). “Everyday experiences of discrimination” was modeled as a continuous variable with scores summed up over each of the eight items on the scale. For categorical analyses, we also created a dichotomous variable for everyday experiences of discrimination, with participants responding to at least one of the eight items with “Rarely”, “Sometimes”, or “Often” categorized as ever having experienced discrimination, and those who responded “Never” to all of the questions categorized as never having discrimination. We then used a test of paired proportions to examine whether the proportions were different between COMPASS I and COMPASS II for each cultural group.

To capture the longitudinal nature of these analyses, and to reduce the potential for reverse causation, worse mental health outcomes in COMPASS II was modeled as a function of discrimination reported in COMPASS I and baseline covariates. 

We first examined the relationships between individual covariates and worse mental health using *t*-tests for continuous variables and chi-squared tests for categorical variables. Then, using binary logistic regression, the first model was the crude model including experiences of discrimination in relation to worse mental health and the individual covariates as separate models. Asian Indians were selected as the reference group for the cultural group analysis, as they were the AAPI group with the lowest level of reported discrimination at baseline. The adjusted model included variables from COMPASS I (baseline), hypothesized to be related to both mental health outcomes and experiences of discrimination. This study also assessed whether the relationship between discrimination and mental health outcomes was modified by cultural group, age, sex, nativity, education, or income. To do this, we created an interaction term between each variable and discrimination, and assessed whether the *p*-value for the interaction was significant (i.e., <0.05).

All statistical tests were 2-sided. The association was considered statistically significant if the 95% confidence interval did not include the null value (corresponding to a *p*-value of 0.05 or less). All assumptions and conditions for binary logistic regression modeling were met. Analyses were conducted using SAS Software, Version 9.4 [35].

## 3. Results

### 3.1. Sample Characteristics

A total of 3177 participants provided complete information for experiences of discrimination and mental health outcomes in COMPASS II (follow-up survey). At the time of the COMPASS I survey, the mean (standard deviation) PHQ-4 score was 2.32 (2.65), with a range of 0–12. A total of 1945 participants (61.2%) were in the normal range, 867 (27.3) with mild mental distress, 252 (7.9%) with moderate mental distress, and 113 (3.6%) with severe mental distress. At the time of the COMPASS II survey, a total of 2056 (64.7%) of participants were in the normal range, 795 (25.0%) with mild, 213 (6.7%) with moderate, and 113 (3.6%) with severe mental distress. Overall, 909 participants (28.6%) experienced worse mental health outcomes from COMPASS I to COMPASS II. 

Participant characteristics for the study sample are shown in Table 1. 

The mean age of 44.6 years (SD = 16.1) was similar to that of COMPASS I, 45.2 years [9]. (SD = 16.4). There were some differences in the distribution of cultural groups, with ethnic Chinese (including persons from China, Hong Kong, and Taiwan; 37.3% vs. 33.9% in COMPASSI) and Native Hawaiian Pacific Islanders; 4.0% vs. 2.3% in COMPASSI) being slightly higher and Koreans (20.6% vs. 22.5% in COMPASSI) being slightly lower. Other sociodemographic variables such as gender, sexual orientation, nativity, and education were similar to COMPASS I with 65.5% females (vs. 64.1%), same percentage of 91.6% participants identifying as having heterosexual orientation, 62.1% being foreign born (vs. 63.4%), and 36.9% with a Bachelor’s degree (vs. 36.1%).

Among participants who completed both surveys, we found that although mental health overall improved among participants who completed the survey, with mean (standard deviation) PHQ-4 score in COMPASS II of 2.13 (2.66) compared to 2.32 (2.65) in COMPASS I, 28.7% of participants reported a worse PHQ-4 score between the surveys. 

In preliminary analyses, using *t*-tests for continuous variables and chi-squared tests for categorical variables, we found that experiences of discrimination, age, marital status, employment status, income, census region, cultural group, and severity of COVID-19 were significantly associated with worse mental health (*p*-value < 0.05 for all), whereas sex, sexual orientation, education, nativity, years in the US, and percent of life in the US were not.

The mean (standard deviation) everyday discrimination score (EDS) overall was 11.49 (4.51) and varied from 9.27 (3.61) for NHPIs to 15.00 (6.27) for Hmong. Table 2 shows the mean score of each of the individual items by cultural group.

**Figure 2:**
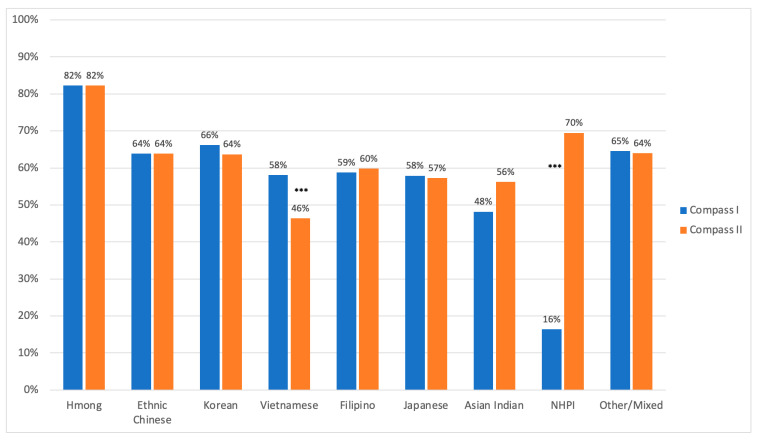
Percentage of participants reporting any experiences of discrimination, Compass I and Compass II. *** Statistically significant.

Over the approximately 1-year period, reported experiences of discrimination remained high in the group, with 60.6% reporting any experiences of discrimination in COMPASS I, compared with 60.2% among the same population in COMPASS II. Within subgroups, experiences were higher for Asian Indians, and Native Hawaiian and Pacific Islanders, while lower for Vietnamese, although the difference was statistically significant only for NHPIs and Vietnamese. The reported experiences were similar for ethnic Chinese, Hmong, and Filipino participants (Figure 2). 

### 3.2. Associations of Discrimination Experience with Mental Health

In the crude model, each unit increase in experiences of discrimination was associated with a 1.03 (95% CI: 1.01–1.04) increase in the odds of worse mental health outcomes (*p* < 0.01). After adjusting for covariates, the relationship was slightly attenuated but remained significant: aOR 1.02 (95% CI 1.01–1.04) (Table 3). Age was also significantly associated with worse mental health outcomes. Relative to those aged 60 and older, those who were younger than 30 years old had 1.70 times (95% CI 1.18–2.45) the odds of reporting worse mental health outcomes. Compared to US born participants, those who had lived in the US for 25% of their life or less were not more likely to report worse mental health outcomes: aOR (95% CI) 1.29 (0.95–1.74); however, those who had lived in the US for >25–50% of their lives or >50–75% of their lives were significantly more likely to report worse mental health outcomes: aORs (95% CI) 1.36 (1.05–1.76) and 1.30 (1.02–1.67), respectively. Cultural group was also associated with worsened mental health outcomes: compared to Asian Indians, Hmong were more likely to report worse mental health outcomes:

aOR (95% CI) vs. Asian Indians: 2.10 (1.01–4.37) as were NHPIs (vs. Asian Indians): 5.09 (2.77–9.36). In post hoc logistic regression analyses, NHPIs were also more likely to report worsened mental health outcomes compared to ethnic Chinese, with aOR of 4.24 (2.69–6.70). 

This study also found that the relationship between experiences of discrimination on mental health varied by cultural group (*p* = 0.02 for interaction between discrimination experiences and cultural group). Due to limited sample sizes of some cultural groups, we stratified the largest groups only: ethnic Chinese, Korean, and Vietnamese. We found that the association was marginally significant among ethnic Chinese and Vietnamese, aOR (95% CI) 1.03 (1.00–1.06) and 1.05 (1.00–1.10), respectively, but not among Koreans: 0.99 (0.95–1.03). Interactions of discrimination with sex, nativity, education, and income were not statistically significant. 

## 4. Discussion

In this longitudinal COVID-19 study, spanning two time points approximately a year apart, we found that while experiences of discrimination remained fairly constant or improved for some groups; two groups, specifically Asian Indians and NHPIs, experienced an increase in discrimination experiences. We also found a modest but significant association between everyday experiences of discrimination and worse mental health outcomes, which persisted after controlling for covariates. Notably, we also observed that the relationship between experiences of discrimination and mental health may vary by cultural group, with a significant relationship found among ethnic Chinese and Vietnamese but not among Koreans. Moreover, we also found that age and percentage of life spent in the United States were significantly related to mental health outcomes. 

The dramatic increase in the reported experiences of discrimination among NHPIs between COMPASS I and COMPASS II is alarming and warrants further attention. Compared to other Asian sub-groups, NPHIs have been disproportionately affected by the COVID-19 pandemic compared to all other AANHPI subgroups [36]. This increase in experiences of discrimination may reflect a reaction to the devastation experienced with NHPI communities as a result of the pandemic. Indeed, in a study of the experiences of discrimination among 252 in the US, Andersen et al. [37] found that NHPIs reported significant instances of discrimination during everyday activities, from 25% of them around obtaining loans to about 52% in public settings. Compounding the increased experiences of discrimination is the observation from several studies that NHPI are less likely to utilize healthcare services because of stigma associated with mental health [38]. While the increase in experiences of discrimination among Asian Indians was not statistically significant, the increase may reflect an upward trend from the start of the pandemic. Studies have suggested that South Asians (who include Asian Indians) are more likely to report psychological distress than non-Hispanic Whites [12]. Taken together, these findings highlight the need to provide culturally responsive [39] interventions that will reduce the mental health disparities of AANHPI [40]. It is also worth noting that while experiences of discrimination among Vietnamese are significantly lower in COMPASS II compared to COMPASS I, nearly 1 in 2 people still report experiences of racism, and as such, outreach efforts to mitigate experiences of racism must continue.

Experiences of discrimination have been linked to mental health outcomes through direct and indirect pathways [41,42]. Discrimination experiences have been shown to increase physiological stress responses, which are linked to increased depression and anxiety [41,43]. In addition, discrimination experiences are also linked indirectly to mental health outcomes through experiences such as mortgage denials, job losses and other traumatic experiences [44,45,46]. 

We found that the association between experiences of discrimination and mental health varied by cultural group. This finding is consistent with observations from Add and colleagues’ study, which were that there were differences in mental health outcomes between South Asian Americans and Whites, but not with East or Southeast Asian Americans compared to Whites [12]. This finding highlights an opportunity for additional programs in Chinese and Vietnamese communities to help moderate the effects of discrimination on mental health. We also found that cultural group was associated with mental health over the one-year period, consistent with the findings from Tiwari et al., that noted differences in the prevalence of psychological distress between East Asians, Southeast Asians, and South Asians. Notably, we observed a relatively high association with worse mental health among Native Hawaiian and Pacific Islanders, consistent with the findings of Subica et al., that identified a heavy burden of anxiety and depression among this group, while being less likely to seek care [47]. This underscores the need for interventions in this community to promote culturally sensitive approaches that maximize service utilization.

In our study, we found that younger age was associated with worse mental health outcomes over time, specifically among the <30-, 30–39-, and 40–49-year age groups. This is consistent with findings from other studies among adults within similar populations [48,49,50,51]. As people age, they may better be able to withstand life experiences and become more resilient, thus reducing the risk of poor mental health [52,53,54]. 

We also found that compared to US-born participants, participants born outside of the US were also more likely to have worsened mental health over time during the COVID-19 pandemic. In addition, the odds increased somewhat with the percentage of life spent in the US, among those who had spent 25- < 50% and 50- < 75% of their lives in the US, compared to US-born. Our findings are consistent with some studies, but not all. In a European study of men across 11 countries, immigrants faced an increased odds of depression even though they had better overall physical health [55]. In a systematic review spanning several European countries, the US, Canada, Australia, New Zealand and Israel, the authors also found that immigrants experienced higher levels of depression and anxiety compared to native-born populations [56]. Conversely, immigrants in the US reported lower odds of mental distress compared to US-US-born individuals [57]. However, we did not find comparable studies that were conducted during the COVID-19 pandemic that examined this association, highlighting the need for more studies, especially among AANHPIs [58]. 

Our study has several key strengths. This is the largest study to our knowledge to disaggregate the experiences of discrimination on mental health among Asian American and Pacific Islander subgroups. As observed in the findings, these populations are heterogeneous, and identifying unique risk profiles in each of these groups can provide better tailored interventions that improve health outcomes in each population. In addition, the longitudinal nature of the study using baseline variables to examine changes over time reduces the opportunity for reverse causation, thus adding more credibility to our findings. Nevertheless, we also acknowledge some important limitations. To begin with, the study used convenience sampling. For example, women were significantly overrepresented compared to men, although we did adjust for sex in the analyses. In addition, since COMPASS I and II were both primarily administered online, access was limited to those who could reliably use the internet. However, we did not find any associations between mental health and education in our crude or adjusted analyses. We also did not collect information on religion, which prevented us from examining the role of religion in mitigating the association between experiences of discrimination and mental health. Finally, the limited representation of some of the subgroups prevented adequate examination in these groups. 

## 5. Conclusions

In sum, in this longitudinal study, experiences of discrimination, cultural group, age, and percentage of life in the United States were associated with worse mental health outcomes over time in this population. Uniquely, we were able to examine these associations during the COVID-19 pandemic. Additional research must be conducted in this sub-groups to examine whether the experiences of discrimination following the pandemic continue to increase for vulnerable groups within this population. Further, public health policies and programs should focus on reducing experiences of discrimination among these groups through both community-based (for example, optimizing community-led responses to crises) and structural initiatives (for example, increasing the number of mental care providers within vulnerable populations) [59], and prioritize efforts to modify the effects of these factors on discrimination, and also address experiences in the effects of discrimination by cultural group. 

## Figures and Tables

**Table 1:** COMPASS study sample characteristics (n = 3177).

Variable		Mental Health after 1 Year		
	Overall (n = 3177)	Worse (n = 909)	Same or Better (n = 2298)	*p*-Value ^2^
Everyday Discrimination Score	11.5 (4.51) ^1^; Range: 8–32	11.86 (4.90) 8–32	11.35 (4.33) 8–32	<0.01
Social and Demographic Factors				
Age (in years)	44.6 (16.1) ^1^; Range: 18–97	42.6 (15.4) 18–86	45.4 (16.3) 18–97	<0.01
<30	739 (23.3)	246 (33.3)	493 (66.7)	<0.01
30–39	567 (17.9)	170 (30.0)	397 (70.0)	
40–49	601 (18.9)	169 (28.1)	432 (71.9)	
50–59	630 (19.8)	181 (28.7)	449 (71.3))	
>60	640 (20.1)	143 (22.3)	497 (77.7)	
Sex				
Female	2081 (65.5)	602 (28.9)	1479 (71.1)	0.71
Male	1069 (33.6)	298 (27.9)	771 (72.1)	
Other/Decline to State	27 (0.9)	9 (33.3)	18 (66.7)	
Sexual Orientation				
Heterosexual	2909 (91.6)	822 (28.3)	2087 (71.7)	0.28
Not Heterosexual	156 (4.9)	53 (34.0)	103 (66.0)	
Other/Decline to State	112 (3.5)	34 (30.4)	78 (69.6)	
Education				0.09
High school or less	494 (15.6)	164 (33.2)	330 (66.8)	
Some college or technical school	338 (10.6)	95 (28.1)	243 (71.9)	
Bachelor’s degree	1147 (36.1)	324 (28.3)	823 (71.8)	
Master’s degree or higher	1159 (36.5)	319 (27.5)	840 (72.5)	
Unknown	39 (1.2)	7 (18.0)	32 (82.0)	
Marital Status				
Single	908 (28.6)	295 (32.5)	613(67.5)	0.01
Married/Living with Partner	2036 (64.1)	558 (27.4)	1478 (72.6)	
Separated/Divorced/Widowed	210 (6.6)	51 (24.3)	159 (75.7)	
Unknown	23 (0.7)	5 (21.7)	18 (78.3)	
Employment Status				
Full–time	1475 (46.4)	428 (29.0)	1047 (71.0)	<0.01
Part–time	541 (17.0)	165 (30.5)	376 (69.5)	
Homemaker	254 (8.0)	65 25.6)	189 (74.4)	
Unemployed	365 (11.5)	125 (34.3)	240 (34.3)	
Retired	342 (10.8)	63 (18.4)	279 (18.4)	
Other/Decline to state	200 (6.3)	63 (31.5)	137 (31.5)	
Annual Household Income ($)				
≤25,000	523 (16.5)	171 (32.7)	352 (67.3)	0.03
>25,000–75,000	883 (27.8)	230 (26.1)	653 (73.9)	
>75,000–150,000	811 (25.5)	250 (30.8)	561 (69.2)	
>150,000	627 (19.8)	167 (26.6)	460 (73.4)	
Decline to state	333 (10.5)	91 (27.3)	242 (72.7)	
Societal Factors				
Census Region				
West	2133 (67.1)	576 (27.0)	1557 (73.0)	0.03 ^3^
Midwest	273 (8.6)	90 (33.0)	183 (67.0)	
Northeast	326 (10.3)	105 (32.2)	221 (67.8)	
South	442 (13.9)	138 (31.2)	304 (68.8)	
Unknown	3 (0.1)	3 (100.0)	0 (0.0)	
Nativity				
US–born	1161 (36.5)	320 (27.6)	841 (72.4)	0.27
Foreign–born	1974 (62.1)	573 (29.0)	1401 (71.0)	
Unknown	42 (1.3)	16 (38.1)	26 (91.9)	
Years in US	24.1 (14.9) ^1^; Range: 0–80	24.1 (15.3) 0–80	24.2 (14.7) 0–76	0.92
Percent of life in the US				
25% or less	331 (13.1)	115 (27.7)	300 (72.3)	0.63
>25 to 50%	588 (18.5)	169 (28.7)	419 (71.3)	
>50 to 75 %	609 (19.2)	177 (29.1)	432 (70.9)	
>75 to 100%	345 (10.9)	112 (32.5)	233 (67.5)	
US Born (100%)	1161 (36.5)	320 (27.6)	841 (72.4)	
Unknown	59 (1.9)	16 (27.1)	43 (72.9)	
Cultural Group				<0.01
Asian Indian	137 (4.3)	31 (22.6)	106 (77.4)	
Ethnic Chinese ^4^	1185 (37.3)	311 (26.2)	874 (73.8)	
Filipino	97 (3.1)	31 (32.0)	66 (68.0)	
Hmong	62 (2.0)	26 (41.9)	36 (58.1)	
Japanese	152 (4.8)	37 (24.3)	115 (75.7)	
Korean	653 (20.6)	194 (29.7)	459 (70.3)	
NHPI ^5^	128 (4.0)	75 (58.6)	53 (41.4)	
Vietnamese	616 (19.4)	152 (24.7)	464 (75.3)	
Other/ Mixed	147 (4.6)	52 (35.4)	95 (64.6)	
The Severity of COVID-19 Where You Live				
A lot less	319 (10.0)	121 (37.9)	198 (62.1)	<0.01
Somewhat less	542 (17.1)	127 (23.4)	415 (76.6)	
About the same	702 (22.1)	202 (28.8)	500 (71.2)	
Somewhat more	901 (28.4)	274 (30.4)	627 (69.6)	
A lot more	700 (22.0)	181 (25.9)	519 (74.1)	
Unknown	13 (0.4)	4 (30.8)	9 (69.2)	

^1^ Mean (standard deviation; SD) for continuous variables ^2^ *p*-values for *t*-tests of continuous variables and chi-squared tests for categorical variables, comparing each variable to change in mental health (same or better versus worse). ^3^ ”Unknown” category was excluded from the chi-squared analysis for this variable due to the small number ^4^ Ethnic Chinese includes mainland Chinese, Hongkonger, Taiwanese, and Huaren. ^5^ Native Hawaiians/Pacific Islanders.

**Table 2:** Experiences of discrimination during the COVID-19 pandemic due to being Asian, Asian American or Pacific Islander by cultural group by mean (standard deviation) and range.

	All (n = 3177)	Asian Indian (n = 137)	Ethnic Chinese ^1^ (n = 1185)	Filipino (n = 97)	Hmong (n = 62)	Japanese (n = 152)	Korean (n = 653)	NHPI ^2^ (n = 128)	Vietnamese (n = 616)	Other/Mixed (n = 147)
	EDS Overall: Total Score for Discrimination Experiences (Sum of EDS_1 through EDS_8)									
Mean (SD) ^3^	11.49 (4.51)	10.89 (4.65)	11.69	11.04 (4.40)	15.00 (6.27)	11.08 (4.45)	11.41 (4.16)	9.27 (3.61)	11.37 (4.52)	12.55 (5.34)
Range	8.00–32.00	8.00–29.00	8.00–29.00	8.00–32.00	8.00–31.00	8.00–29.00	8.00–32.00	8.00–28.00	8.00–32.00	8.00–30.00
	EDS_1: … you been treated with less respect than other people…									
Mean (SD)	1.72 (0.81)	1.52 (0.81)	1.76 (0.79)	1.59 (0.79)	2.10 (0.90)	1.66 (0.77)	1.79 (0.81)	1.23 (0.67)	1.68 (0.83)	1.92 (0.88)
Range	1.00–4.00	1.00–4.00	1.00–4.00	1.00–4.00	1.00–4.00	1.00–4.00	1.00–4.00	1.00–4.00	1.00–4.00	1.00–4.00
	EDS_2: … you been treated unfairly at restaurants or stores…									
Mean (SD)	1.45 (0.72)	1.35 (0.70)	1.47 (0.70)	1.37 (0.70)	1.81 (0.90)	1.40 (0.70)	1.45 (0.72)	1.16 (0.62)	1.46 (0.76)	1.61 (0.52)
Range	1.00–4.00	1.00–4.00	1.00–4.00	1.00–4.00	1.00–4.00	1.00–4.00	1.00–4.00	1.00–4.00	1.00–4.00	1.00–4.00
	EDS_3: … people criticized your accent or the way you speak…									
Mean (SD)	1.35 (0.68)	1.35 (0.66)	1.36 (0.67)	1.29 (0.74)	1.50 (0.82)	1.23 (0.64)	1.39 (0.69)	1.13 (0.48)	1.36 (0.70)	1.41 (0.75)
Range	1.00–4.00	1.00–4.00	1.00–4.00	1.00–4.00	1.00–4.00	1.00–4.00	1.00–4.00	1.00–4.00	1.00–4.00	1.00–4.00
	EDS_4: … people acted as if they think you are not smart…									
Mean (SD)	1.33 (0.66)	1.31 (0.66)	1.33 (0.62)	1.35 (0.71)	1.87 (0.95)	1.24 (0.62)	1.32 (0.64)	1.19 (0.57)	1.35 (0.68)	1.41 (0.77)
Range	1.00–4.00	1.00–4.00	1.00–4.00	1.00–4.00	1.00–4.00	1.00–4.00	1.00–4.00	1.00–4.00	1.00–4.00	1.00–4.00
	EDS_5: … people acted as if they are afraid of you…									
Mean (SD)	1.48 (0.74)	1.27 (0.62)	1.53 (0.74)	1.36 (0.68)	2.13 (1.00)	1.47 (0.75)	1.45 (0.71)	1.13 (0.46)	1.46 (0.73)	1.65 (0.87)
Range	1.00–4.00	1.00–4.00	1.00–4.00	1.00–4.00	1.00–4.00	1.00–4.00	1.00–4.00	1.00–4.00	1.00–4.00	1.00–4.00
	EDS_6: … people acted as if they think you are dishonest…									
Mean (SD)	1.27 (0.60)	1.28 (0.62)	1.29 (0.58)	1.18 (0.50)	1.68 (0.90)	1.22 (0.57)	1.21 (0.52)	1.13 (0.43)	1.29 (0.64)	1.41 (0.77)
Range	1.00–4.00	1.00–4.00	1.00–4.00	1.00–4.00	1.00–4.00	1.00–4.00	1.00–4.00	1.00–4.00	1.00–4.00	1.00–4.00
	EDS_7: … people acted as if they’re better than you are…									
Mean (SD)	1.55 (0.81)	1.54 (0.84)	1.55 (0.77)	1.60 (0.89)	2.24 (1.10)	1.53 (0.85)	1.55 (0.78)	1.21 (0.62)	1.50 (0.78)	1.72 (0.93)
Range	1.00–4.00	1.00–4.00	1.00–4.00	1.00–4.00	1.00–4.00	1.00–4.00	1.00–4.00	1.00–4.00	1.00–4.00	1.00–4.00
	EDS_8: … you been threatened or harassed…									
Mean (SD)	1.33 (0.64)	1.28 (0.59)	1.41 (0.69)	1.31 (0.64)	1.65 (0.77)	1.34 (0.71)	1.25 (0.55)	1.10 (0.41)	1.26 (0.58)	1.43 (0.74)
Range	1.00–4.00	1.00–3.00	1.00–4.00	1.00–4.00	1.00–3.00	1.00–4.00	1.00–4.00	1.00–3.00	1.00–4.00	1.00–4.00

^1^ Ethnic Chinese includes mainland Chinese, Hongkonger, Taiwanese, and Huaren. ^2^ Native Hawaiians/Pacific Islanders. ^3^ SD = standard deviation

**Table 3:** Odds Ratios and 95% Confidence Intervals for Worse Mental Health (Yes vs. No).

Variable	Crude Model	Adjusted Model ^1^
Everyday Discrimination	1.03 (1.01–1.04)	1.02 (1.01–1.04)
*p*-value ^2^	<0.01	0.01
Social and Demographic Factors		
Age (in years)		
<30	1.73 (1.36–2.21)	1.70 (1.18–2.44)
30–39	1.49 (1.15–1.93)	1.48 (1.05–2.08)
40–49	1.36 (1.05–1.76)	1.40 (1.00–1.95)
50–59	1.40 (1.09–1.81)	1.28 (0.94–1.75)
>60	Reference	Reference
*p*-value	<0.01	0.07
Sex		
Female	Reference	Reference
Male	0.95 (0.81–1.12)	0.97 (0.81–1.15)
Other/Decline to state	1.23 (0.55–2.75)	1.01 (0.41–2.45)
*p*-value	0.71	0.93
Sexual Orientation		
Heterosexual	Reference	Reference
Not Heterosexual	1.31 (0.93–1.84)	1.16 (0.80–1.68)
Decline to State	1.11 (0.73– 1.67)	1.15 (0.74–1.80)
*p*-value	0.28	0.63
Education		
High school or less	Reference	Reference
Some college or technical school	0.79 (0.58–1.06)	0.80 (0.58–1.12)
Bachelor’s degree	0.79 (0.63–0.99)	0.88 (0.67–1.16)
Master’s degree or higher	0.76 (0.61–0.96)	0.88 (0.66–1.17)
Unknown	0.44 (0.19–1.02)	0.56 (0.23–1.36)
*p*-value	0.09	0.57
Marital Status		
Single	1.28 (1.08–1.51)	1.17 (0.93–1.48)
Married/Living with Partner	Reference	Reference
Separated/Divorced/Widowed	0.85 (0.61–1.18)	0.83 (0.58–1.20)
Unknown	0.74 (0.27–1.99)	0.73 (0.26–2.09)
*p*-value	0.01	0.33
Employment Status		
Full–time	Reference	Reference
Part–time	1.07 (0.87–1.33)	1.02 (0.81–1.30)
Homemaker	0.84 (0.62–1.14)	0.87 (0.63–1.22)
Unemployed	1.27 (1.00–1.63)	1.10 (0.84–1.44)
Retired	0.55 (0.41–0.74)	0.82 (0.56–1.20)
Other/Decline to state	1.13 (0.82–1.55)	1.12 (0.80–1.59)
*p*-value	<0.01	0.69
Annual Household Income ($)		
≤25,000	Reference	Reference
>25,000–75,000	0.73 (0.57–0.92)	0.79 (0.60–1.04)
>75,000–150,000	0.92 (0.73–1.16)	1.08 (0.80–1.45)
>150,000	0.75 (0.58–0.96)	0.98 (0.70–1.36)
Decline to state	0.77 (0.57–1.05)	1.00 (0.71–1.43)
*p*-value	0.03	0.08
Societal Factors		
Census Region		
West	Reference	Reference
Midwest	1.33 (1.02–1.74)	1.14 (0.83–1.57)
Northeast	1.28 (1.00–1.65)	1.27 (0.97–1.66)
South	1.23 (0.98–1.53)	1.17 (0.92–1.49)
*p*-value	0.07	0.41
Percent of life in the US		
25% or less	1.01 (0.78–1.29)	1.29 (0.95–1.74)
>25 to 50%	1.06 (0.85–1.32)	1.36 (1.04–1.76)
>50 to 75%	1.08 (0.87–1.34)	1.30 (1.02–1.67)
>75 to 100%	1.26 (0.98–1.64)	1.27 (0.94–1.68)
US Born (100%)	Reference	Reference
Unknown	0.98 (0.54–1.76)	1.11 (0.60–2.05)
*p*-value	0.64	0.21
Cultural Group		
Asian Indian	Reference	Reference
Ethnic Chinese ^1^	1.22 (0.80–1.85)	1.20 (0.78–1.85)
Filipino	1.61 (0.90–2.88)	1.62 (0.88–2.98)
Hmong	2.47 (1.30–4.70)	2.10 (1.01–4.37)
Japanese	1.10 (0.64–1.90)	1.50 (0.85–2.67)
Korean	1.45 (0.94–2.23)	1.31 (0.84–2.06)
NHPI	4.84 (2.84–8.24)	5.09 (2.77–9.36)
Vietnamese	1.12 (0.72–1.74)	1.12 (0.71–1.79)
Other/Mixed	1.83 (1.09–3.06)	1.75 (1.01–3.03)
*p*-value	<0.01	<0.01
The Severity of COVID-19 Where You Live		
A lot less	Reference	Reference
Somewhat less	0.50 (0.37–0.68)	0.71 (0.50–1.00)
About the same	0.66 (0.50–0.87)	0.84 (0.60–1.16)
Somewhat more	0.72 (0.55–0.93)	0.95 (0.70–1.31)
A lot more	0.57 (0.43–0.76)	0.75 (0.54–1.04)
*p*-value	<0.01	0.12

^1^ Adjusted model adjusted for cultural group, sex, sexual orientation, age, percent of life in the US, marital status, employment status, education, annual household income, census region, and severity of COVID-19 where they live. ^2^ *p*-values noted for the significance of each variable (for the single variable for continuous, and across categories for categorical variables).

## Data Availability

Data for the study is available upon request.
